# Wafer-Scale Fabrication of Uniform Few-Layer Hexagonal Boron Nitride Stacks for Memristor Applications

**DOI:** 10.3390/nano16100611

**Published:** 2026-05-16

**Authors:** Jiawei Wu, Jiahao Wang, Qinci Wu, Bingchen Han, Mengwei Li, Junqiang Wang, Hongtao Liu

**Affiliations:** 1 Academy for Advanced Interdisciplinary Research, School of Instrument and Electronics, North University of China, Taiyuan 030051, China; sz202306175@st.nuc.edu.cn (J.W.); lmw@nuc.edu.cn (M.L.); 2 Beijing Graphene Institute, Beijing 100095, China; 3 Center for Nanochemistry, Beijing Science and Engineering Center for Nanocarbons, Beijing National Laboratory for Molecular Sciences, College of Chemistry and Molecular Engineering, Peking University, Beijing 100871, China; wangjh-cnc@stu.pku.edu.cn (J.W.); wuqc-cnc@pku.edu.cn (Q.W.); hanbc-cnc@stu.pku.edu.cn (B.H.); 4 Academy for Advanced Interdisciplinary Studies, Peking University, Beijing 100871, China

**Keywords:** few-layer hexagonal boron nitride, ultraflat single-crystal hBN, dry lamination, memristor

## Abstract

Few-layer hexagonal boron nitride (hBN) is a promising two-dimensional dielectric for electronic and neuromorphic devices. However, its practical deployment is often hindered by the thickness nonuniformity of as-grown samples and by defects introduced during the transfer-stacking process of assembled samples. In particular, the influence of the initial hBN quality on the final stacked-film quality remains insufficiently understood. Here, we report a wafer-scale strategy for fabricating high-quality few-layer hBN based on ultraflat single-crystal hBN (USC-hBN) monolayers. Compared with transfer-stacked hBN grown on Cu foil (rough hBN), stacked few-layer USC-hBN shows a much lower surface roughness and a drastically reduced wrinkle density, indicating superior flatness and interfacial cleanliness. Furthermore, memristors fabricated from six-layer USC-hBN exhibit clearer resistive-switching behavior and a higher ON/OFF ratio than those based on rough hBN, owing to the more uniform surface/interface. These results demonstrate that source-material flatness is a critical determinant of transfer-stacked hBN quality and device performance. This work provides an effective route toward reliable integration of high-quality two-dimensional dielectric films.

## 1. Introduction

Few-layer hexagonal boron nitride (FL-hBN) exhibits thickness-dependent properties that distinguish it from both monolayer and bulk hBN. Compared with monolayer hBN, FL-hBN offers stronger dielectric screening, more robust tunneling-barrier behavior, and thickness-dependent interlayer coupling, all of which are highly relevant to electronic transport and interfacial regulation [1,2]. In addition, FL-hBN combines excellent electronic, thermal and chemical stability with high breakdown strength, mechanical robustness, optical transparency, and efficient heat dissipation, making it highly attractive for high-performance electronic, optoelectronic, and neuromorphic devices [3,4,5].

Among the available approaches for constructing FL-hBN, transfer and stacking of chemical vapor deposition (CVD) grown monolayer films provides a practical route to controlled thickness while maintaining compatibility with diverse target substrates [6,7]. However, transfer-stacked hBN frequently suffers from wrinkles, cracks, contamination, and thickness nonuniformity, which can undermine film integrity and device reliability [8,9]. Such structural imperfections not only degrade the physical continuity of the film but also introduce localized electrical inhomogeneities, which are particularly detrimental for applications involving tunneling transport or resistive switching [10,11]. Although these issues are commonly attributed to the transfer process itself, the influence of the initial growth substrate on the final quality of stacked hBN films remains insufficiently understood. Recent studies further indicate that both the intrinsic quality of CVD-grown hBN and the preservation of its crystallinity during transfer are crucial to the structural quality of the assembled films [12,13].

To date, most CVD-grown hBN films have been synthesized on polycrystalline Cu foils, where surface roughness, step bunching, and grain boundaries can introduce structural inhomogeneity into the as-grown layers [14,15]. Such initial imperfections may be further amplified during transfer and stacking, while even layer-selective growth strategies still require precise control over thickness uniformity and substrate quality [16]. In parallel, substantial progress has been achieved in the wafer-scale growth of single-crystal hBN, including self-collimated grain growth, epitaxial monolayer growth on Cu, and epitaxial multilayer growth on Ni(111) [17,18,19,20]. More recently, submicron-spacing vapor deposition and ultraflat single-crystal hBN wafer have further expanded the materials platform for obtaining high-quality large-area films [21,22]. These advances provide a promising foundation for constructing transfer-stacked FL-hBN with greatly improved structural quality.

In this work, we develop a transfer-stacking approach for wafer-scale uniform FL-hBN films and demonstrate that the quality of the starting materials critically governs the quality of the resulting stacks. By employing a dry transfer strategy designed to suppress transfer-induced defects, we show that ultraflat single-crystal hBN (USC-hBN) yields stacked films with low wrinkle density, reduced surface roughness, and improved large-area uniformity. Memristors based on six-layer hBN fabricated from USC-hBN monolayers exhibit clearer resistive-switching characteristics and a higher ON/OFF ratio than those fabricated from rough hBN monolayers grown on conventional Cu foil (rough hBN), indicating improved conductive-filament controllability and device stability. These results reveal the critical role of source hBN quality in transfer-stacked structures and highlight its importance for the development of reliable two-dimensional dielectric-based electronic devices.

## 2. Materials and Methods

### 2.1. Fabrication of Few-Layer hBN

CVD-grown USC-hBN on CuNi(111)/sapphire substrates and rough hBN grown on Cu foils were used as the starting materials; the corresponding growth parameters have been described elsewhere [22]. The fabrication process for few-layer hBN is identical for both materials. Taking USC-hBN as an example, the detailed fabrication steps are as follows: A borneol layer was first spin-coated onto hBN/CuNi(111)/sapphire at 1000 rpm for 1 min from an isopropyl alcohol solution of borneol (25 wt%, >97% purity, Alfa Aesar, Shanghai, China), followed immediately by spin-coating of a PPC layer at 1000 rpm for 1 min from 0.1 g mL^−1^ anisole solution of poly(propylene carbonate) (PPC, *M*_w_ = 200,000, Empower Materials, New Castle, DE, USA). The sample was then baked at 80 °C for 5 min. Subsequently, a poly(methyl methacrylate) (PMMA) layer (950K A4, Xi’an Boyan Micro-nano Informational Technology Co., Ltd., Xi’an, China) was spin-coated at 1000 rpm for 1 min and baked at 150 °C for 5 min, resulting in a PMMA/PPC/borneol/hBN/CuNi(111)/sapphire composite structure. The resulting composite structure was then immersed in a 1 mol/L (NH)_4_S_2_O_8_ (Rhawn, Shanghai Yien Chemical Technology Co., Ltd., Shanghai, China) to etch away the CuNi film. Following etching, the sample was rinsed with deionized water to remove the residual etchant, and the PMMA/PPC/borneol/hBN film was transferred onto a SiO_2_/Si substrate and then dried in a fume hood. After drying, a polydimethylsiloxane (PDMS) sheet (WF-40×40-0060-X4, Gel-Pak, Hayward, CA, USA) was laminated onto the PMMA surface using a commercial laminator (LM-330ID, Rayson Co., Ltd., Foshan, China). The resulting PDMS/PMMA/PPC/borneol/hBN composite film was subsequently immersed in deionized water, where water intercalated into the interface between hBN and the Si substrate owing to the hydrophilic surface of SiO_2_/Si, thereby enabling the composite film to detach from the SiO_2_/Si substrate. After the composite film was dried, it was laminated onto another clean hBN wafer. The sample was then baked at 180 °C for 5 min to release the upper PDMS layer and promote uniform contact between the hBN layers, thereby yielding bilayer hBN. The above procedure was repeated according to the desired number of layers, and the stacked hBN was finally transferred onto a target substrate, such as a SiO_2_/Si substrate. To further enhance the interaction between the few-layer hBN and the substrate, the sample was baked at 180 °C for 2 h. Subsequently, PMMA, PPC, and borneol were removed using acetone vapor, resulting in few-layer hBN on the SiO_2_/Si substrate.

### 2.2. Fabrication of Memristors Based on Few-Layer hBN

A layer of LOR5B photoresist was first spin-coated onto a SiO_2_/Si substrate and baked at 180 °C for 3 min. Subsequently, S1813 photoresist was spin-coated on top of it and baked at 110 °C for 2 min. The photoresist was then exposed using an MLA500 maskless laser direct-writing system, followed by development in TMAH and rinsing with deionized water to define the bottom-electrode pattern. A 20 nm Au bottom electrode was deposited using a DE400 electron-beam evaporation system, and the bottom electrode on the SiO_2_/Si substrate was obtained after a lift-off process. Six-layer hBN was then transferred onto the bottom electrode. The above photolithography and lift-off procedures were repeated, followed by the deposition of 5 nm Ag and 50 nm Au as the top electrode, completing the fabrication of the memristor device.

### 2.3. Characterization

The surface morphology of hBN was characterized using a Nikon Olympus LV100ND optical microscope (Tokyo, Japan), a BW-S501 white-light interferometric optical profiler (Tokyo, Japan), and a Dimension ICON atomic force microscope (Santa Barbara, CA, USA). Raman characterization of few-layer hBN was performed using a LabRAM HR Evolution Raman spectrometer (Paris, France) with 532 nm laser. The surface morphology of the transferred bilayer hBN was further examined using an FEI Quattro S field-emission scanning electron microscope (Hillsboro, OR, USA). Trans-mission electron microscopy images were acquired using a JEM-ARM200F microscope (Tokyo, Japan). Finally, the electrical characteristics of the memristors were measured using a B1500A semiconductor parameter analyzer (Santa Rosa, CA, USA) equipped with a CINDBEST CH-8 probe station (Shenzhen, China).

## 3. Results and Discussion

### 3.1. Comparison of Source hBN Materials

**Figure 1:**
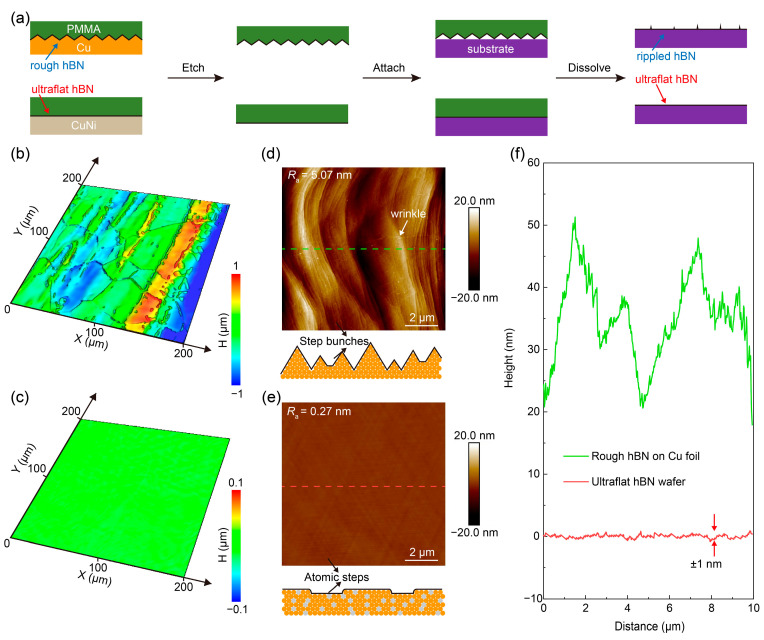
Growth-substrate-dependent flatness of monolayer hBN and its influence on transfer samples. (**a**) Schematic illustration of the transfer process of hBN grown on rough and flat metal substrates. (**b**) Large-area surface topography maps of hBN grown on Cu foil. (**c**) Large-area surface topography map of hBN grown on CuNi (111)/sapphire. (**d**) Representative AFM image of hBN grown on Cu foil. (**e**) Representative AFM image of hBN grown on CuNi (111)/sapphire. (**f**) Height profiles along the lines in (**d**,**e**).

The surface morphology of monolayer hBN grown on different substrates largely determines the quality of the film after transfer onto a target substrate. Recent studies have shown that flatter growth surfaces generally yield transferred hBN films with lower wrinkle density and reduced surface roughness [23]. As illustrated in Figure 1a, a polymer support layer, such as PMMA, is commonly used to transfer hBN onto a target substrate. However, the periodic step-like topography of the PMMA/hBN stack is often mismatched with a flat target surface, resulting in nanoscale ripples after removal of the PMMA layer. In contrast, flatter hBN better preserves its structural integrity and surface flatness during transfer. Conventional hBN films grown by CVD on Cu foil often deviate markedly from the ideal flat morphology, as shown in Figure 1b. Its morphology is severely restricted by the inherent roughness and surface steps of commercial Cu foils, which can promote wrinkle formation and degrade film uniformity during growth and subsequent transfer (Figure 1d). These surface irregularities lead to pronounced height variations in the hBN film, which can reach tens of nanometers even over lateral distances of only a few micrometers (Figure 1f and Figure S1). As a result, the formation of structural wrinkles becomes nearly inevitable when transferring these conventionally rough hBN films onto target substrates.

Guided by this understanding, CuNi(111)/sapphire was adopted as the growth substrate in place of conventional Cu foil, yielding hBN films with a much flatter surface (Figure 1c). This improvement arises from the strong hBN–CuNi(111) interaction, which suppresses the formation of grain boundaries and wrinkles during growth [22], thereby enabling atomically flat hBN films (Figure 1e). Consequently, the step-height variation across the wafer-scale hBN is reduced to ~1 nm (Figure 1f and Figure S2), markedly lower than the much larger height fluctuations observed in rough hBN.

### 3.2. Transfer of Few-Layer hBN

**Figure 2:**
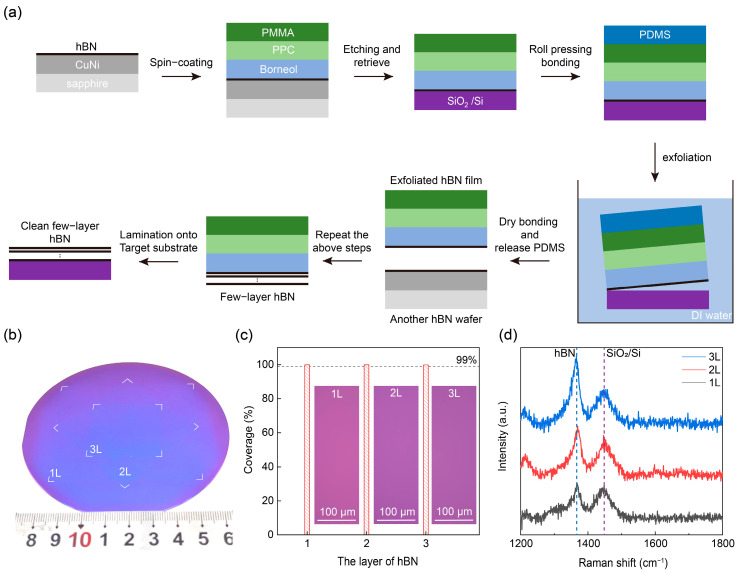
Dry transfer-stacking wafer-scale few-layer USC-hBN and its structural characterization. (**a**) Schematic illustration of the layer-by-layer dry transfer process using a PMMA/PPC/borneol transfer medium supported by PDMS, followed by sequential stacking and final lamination onto the target substrate. (**b**) Photograph of wafer-scale stacked few-layer USC-hBN on a SiO_2_/Si substrate. (**c**) Histograms of coverage of transfer-stacked monolayer (1 L) to trilayer (3 L) USC-hBN. Inset: Optical microscopy images of USC-hBN with various layer numbers. (**d**) Raman spectra of transfer-stacked monolayer to trilayer USC-hBN.

A critical prerequisite for obtaining clean interfaces in stacked hBN is to ensure interfacial cleanliness and dry prior to lamination. Any residual solvent or contamination trapped at the interface can lead to bubble formation and interfacial defects, which deteriorate both structural integrity and electrical performance. To achieve high-quality layer-by-layer stacking, a multilayer transfer medium was designed to enable the sequential release and dry assembly of monolayer hBN (Figure 2a). This strategy avoids the use of liquid at the stacking stage, thereby minimizing the introduction of contaminants and interfacial residues. The combination of PDMS, PMMA, PPC, and borneol as the transfer medium enabled the intact, contamination-free transfer of wafer-scale hBN. Specifically, PDMS serves as a flexible carrier, PMMA as a temporary support, and PPC as a thermally tunable adhesion layer for conformal contact and film release. Borneol acts as a volatile spacer to reduce polymer residue and interfacial contamination [8,23].

Using the designed transfer strategy, large-area few-layer USC-hBN with clean interfaces was successfully stacked and transferred onto SiO_2_/Si substrates (Figure 2b). Optical micrographs of the stacked hBN reveal distinct and uniform contrast variations corresponding to different layer numbers, suggesting high thickness homogeneity and clean interfaces across the whole film (in set of Figure 2c and Figure S3). Notably, the transferred films are free of visible contaminants, and optical characterization confirms a coverage exceeding 99%. This underscores the exceptional integrity and uniformity of the stacking process. Furthermore, Raman spectroscopy (Figure 2d) shows that the signal intensity scales linearly with the layer number, confirming that the intrinsic high quality of the wafer-scale grown hBN is preserved after transfer and stacking.

### 3.3. Characterization of Transferred Bilayer hBN

**Figure 3:**
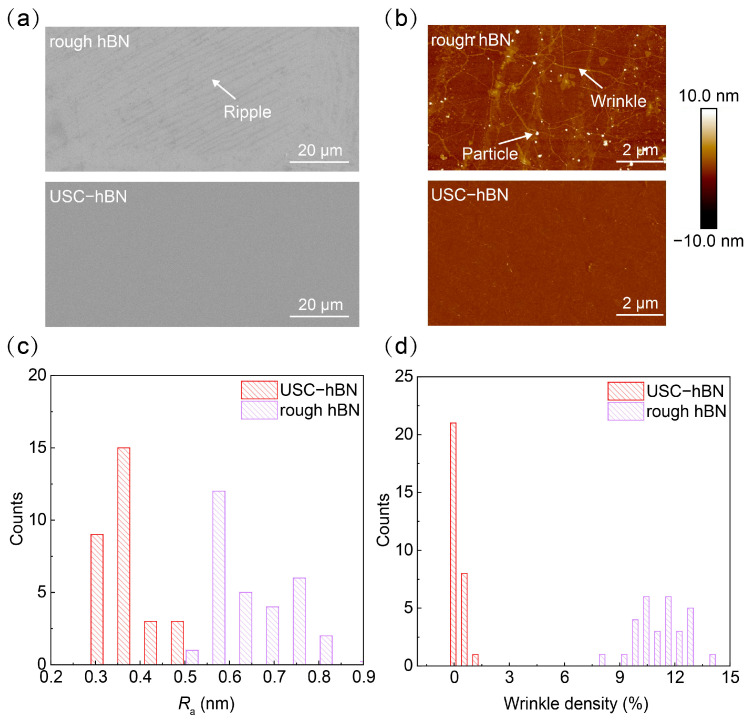
Surface quality comparison between stacked bilayer USC-hBN and bilayer rough hBN after transfer. (**a**) SEM images of bilayer rough hBN and bilayer USC-hBN. (**b**) AFM images of bilayer rough hBN and bilayer USC-hBN. (**c**) Histograms of surface roughness of bilayer rough hBN and USC-hBN extracted from AFM images. (**d**) Histograms of wrinkle density of bilayer rough hBN and USC-hBN extracted from AFM images.

Benefiting from both the superior source material and the optimized transfer process, as-stacked USC-hBN exhibits a much flatter surface after transfer than that derived from rough hBN, as exemplified by the bilayer case (Figure 3a,b). In contrast, the rough hBN sample shows numerous wrinkles (Figure S4), which originate from the mismatch between the rough initial morphology and the flat target substrate during transfer. The surface roughness (*R*_a_) of stacked rough hBN is ~0.67 nm (Figure 3c), while that of stacked USC-hBN is significantly reduced to ~0.35 nm. This nearly twofold reduction in roughness reflects the effective preservation of the intrinsic flatness of USC-hBN throughout the transfer-stacking process. Likewise, the wrinkle density of stacked USC-hBN is only 0.33%, far lower than the 11.5% measured for stacked rough hBN (Figure 3d). Together, these results confirm the superior surface cleanliness, flatness, and uniformity of stacked USC-hBN. Such improvements are expected to significantly influence interface quality and local electric-field distribution, both of which are highly sensitive to nanoscale surface fluctuations.

### 3.4. Impact of hBN Film Quality on Memristor Performance

**Figure 4:**
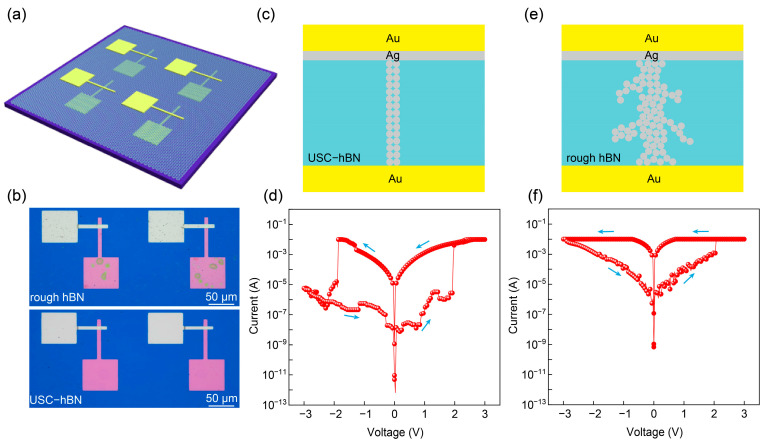
Memristors based on stacked few-layer USC-hBN and rough hBN. (**a**) Schematic diagram of few-layer hBN memristor array. (**b**) Optical microscopy images of memristors fabricated using six-layer USC-hBN and rough hBN. (**c**) Schematic diagram of conductive filament formation in the memristor based on six-layer USC-hBN. (**d**) Representative I–V curve of the memristor based on six-layer USC-hBN. (**e**) Schematic diagram of conductive filament formation in the memristor based on six-layer rough hBN. (**f**) Representative I–V curve of the memristor based on six-layer rough hBN. The arrows indicate the direction of the voltage sweep.

To further demonstrate the advantages of stacked FL-hBN for device applications, we fabricated memristors using few-layer USC-hBN and rough hBN as memristive layers, respectively (Figure 4a). Specifically, a thickness of six layers was chosen as a practical compromise between dielectric reliability and switching accessibility (Figure S5). This thickness ensures sufficient insulation to suppress premature leakage while still allowing the formation of conductive filaments under an applied electric field, thereby enabling observable resistive-switching behavior. Optical microscopy images (Figure 4b) show that the device based on six-layer USC-hBN exhibits a cleaner and more uniform surface (Figure S6). Such improved surface homogeneity indicates reduced contamination and fewer structural imperfections, which are essential for achieving reproducible electrical characteristics across large-area devices. As illustrated in Figure 4c, the six-layer USC-hBN memristor may enable relatively finer and more controllable conductive pathway evolution. We hypothesize that this behavior may be associated with the cleaner and more uniform surface/interface of USC-hBN, which could suppress local electric-field fluctuations induced by defects, residual contamination, and interfacial irregularities, thereby favoring a more regulated evolution of conductive pathways [24,25]. Accordingly, its representative *I*–*V* curve (Figure 4d) exhibits binary resistive-switching characteristics and a high ON/OFF ratio, suggesting improved switching controllability. To further compare the device performance with previous studies, a benchmark comparison with representative hBN-based memristors is provided in Table S1, where the hBN type, thickness, wafer size, and ON/OFF ratio are summarized. In contrast, the memristor based on six-layer rough hBN is likely to experience more nonuniform local electric-field distribution arising from surface topographical fluctuations and structural defects (Figure 4e). Such inhomogeneity may induce disordered conduction and the gradual formation of randomly distributed conductive filaments, ultimately degrading device performance. Moreover, the presence of residual contamination and interfacial gaps may further contribute to local field concentration, accelerating uncontrolled filament growth and rupture. As a result, its representative *I*–*V* curve (Figure 4f) shows a lower ON/OFF ratio and a continuum of resistance states, indicative of analog switching. It should be noted that the above interpretation is based primarily on the morphological differences in hBN films and representative electrical characteristics. A definitive identification of the conduction mechanism, such as Poole–Frenkel emission, Schottky emission, tunneling, or space-charge-limited conduction, would require further transport-model fitting and temperature-dependent electrical measurements. Therefore, the assignment of the switching behavior to a specific conduction mechanism still requires further investigation. These results demonstrate that the surface and interfacial quality of hBN films can strongly influence conductive pathway evolution and memristor performance.

Although the present memristor results demonstrate the influence of stacked hBN surface/interface quality on representative resistive-switching behavior, the devices have not yet been optimized for comprehensive reliability evaluation. In particular, long-cycle endurance, and long-term retention characteristics are not included in the current study. Further optimization of electrode patterning, metal deposition, interfacial cleanliness, and overall device fabrication will be required to evaluate the device-level uniformity and reliability.

## 4. Conclusions

In summary, this work demonstrates a wafer-scale fabrication of high-quality few-layer hBN based on USC-hBN. Using ultraflat hBN as the starting material, the as-stacked FL-hBN exhibits greatly suppressed height fluctuation and markedly improved surface flatness. A dry, layer-by-layer transfer strategy using PDMS/PMMA/PPC/borneol as transfer medium enables the clean stacking of monolayer hBN into large-area FL-hBN with preserved crystallinity, high coverage, and clean interfaces. Compared with transfer-stacked rough hBN, stacked USC-hBN shows smaller surface roughness and dramatically reduced wrinkle density, confirming its superior surface uniformity and cleanliness. Memristors fabricated from six-layer USC-hBN display clearer resistive-switching characteristics and a higher ON/OFF ratio than those based on rough hBN, owing to the more uniform surface/interface. These results establish source-material flatness as a critical factor governing the quality of transfer-stacked FL-hBN and its potential influence on device switching behavior. Further studies are needed to optimize the device fabrication process and systematically investigate the device uniformity and reliability based on the stacked FL-hBN. This work provides an effective strategy for integrating high-quality two-dimensional dielectric films into future electronic and neuromorphic devices.

## Data Availability

The original contributions presented in the study are included in the article and supplementary materials. Further inquiries can be directed to the corresponding author.

## Supplementary Materials

The following supporting information can be downloaded at: https://www.mdpi.com/article/10.3390/nano16100611/s1. Figure S1: Rough hBN film on the copper foil; Figure S2: Ultraflat single-crystal hBN on the CuNi(111)/sapphire wafer; Figure S3: Optical microscopy images of the stacked few-layer USC-hBN; Figure S4: AFM images of the stacked bilayer hBN; Figure S5: Cross-sectional TEM image of stacked six-layer USC-hBN; Figure S6: AFM images of stacked six-layer hBN during memristor fabrication; Table S1. Benchmark comparison of representative hBN-based memristors. References [4,24,26,27] are cited in the supplementary materials.

## Abbreviations

The following abbreviations are used in this manuscript:

hBN	Hexagonal boron nitride
FL-hBN	Few-layer hexagonal boron nitride
USC-hBN	Ultraflat single-crystal hexagonal boron nitride
Rough hBN	Hexagonal boron nitride grown on Cu foil
CVD	Chemical vapor deposition
AFM	Atomic force microscope
*I*-*V*	Current–voltage
