# Supplementary material for: Wafer-Scale Fabrication of Uniform Few-Layer Hexagonal Boron Nitride Stacks for Memristor Applications
Source: Nanomaterials (Basel). 2026 May 16;16(10):611. doi: 10.3390/nano16100611 (PMC13209888; doi:10.3390/nano16100611)
Supplement: Supplementary file 1 [file nanomaterials-16-00611-s001.zip › nanomaterials-4317444-supplementary.pdf]

Supplementary Materials

Figure. S1 Rough hBN film on the copper foil.
Figure. S2 Ultraflat single-crystal hBN on the CuNi(111)/sapphire wafer.
Figure. S3 Optical microscopy images of the stacked few-layer USC-hBN.
Figure. S4 AFM images of the stacked bilayer hBN
Figure. S5 Cross-sectional TEM image of stacked six-layer USC-hBN.
Figure. S6 AFM images of stacked six-layer hBN during memristor fabrication.
Table. S1 Benchmark comparison of representative hBN-based memristors.

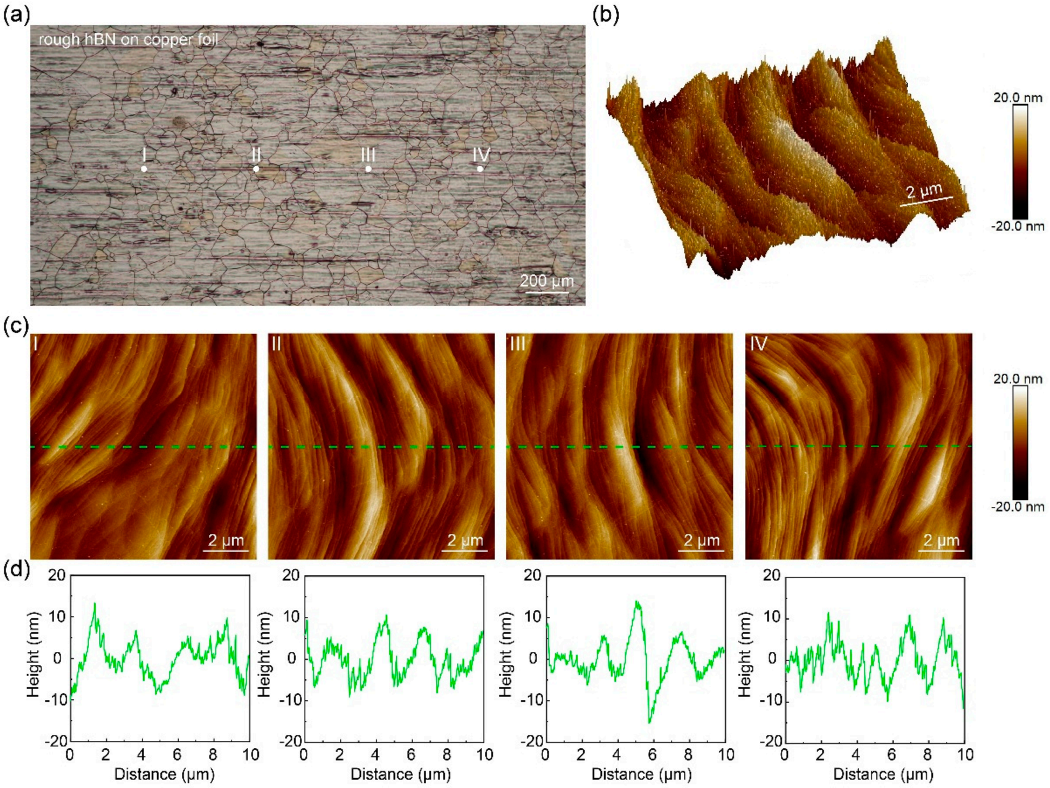

Figure. S1 Rough hBN film on the copper foil. (a) Optical microscopy image of rough hBN film on the copper foil. (b) 3D AFM image of rough hBN film on the copper foil. (c) Typical AFM images revealing dense step bunches on the hBN surface from the marked regions in (a). (d) Height profiles along the green lines in (c),demonstrating the height differences of step bunches can reach several tens of nanometers at a lateral scale of 10 μm.

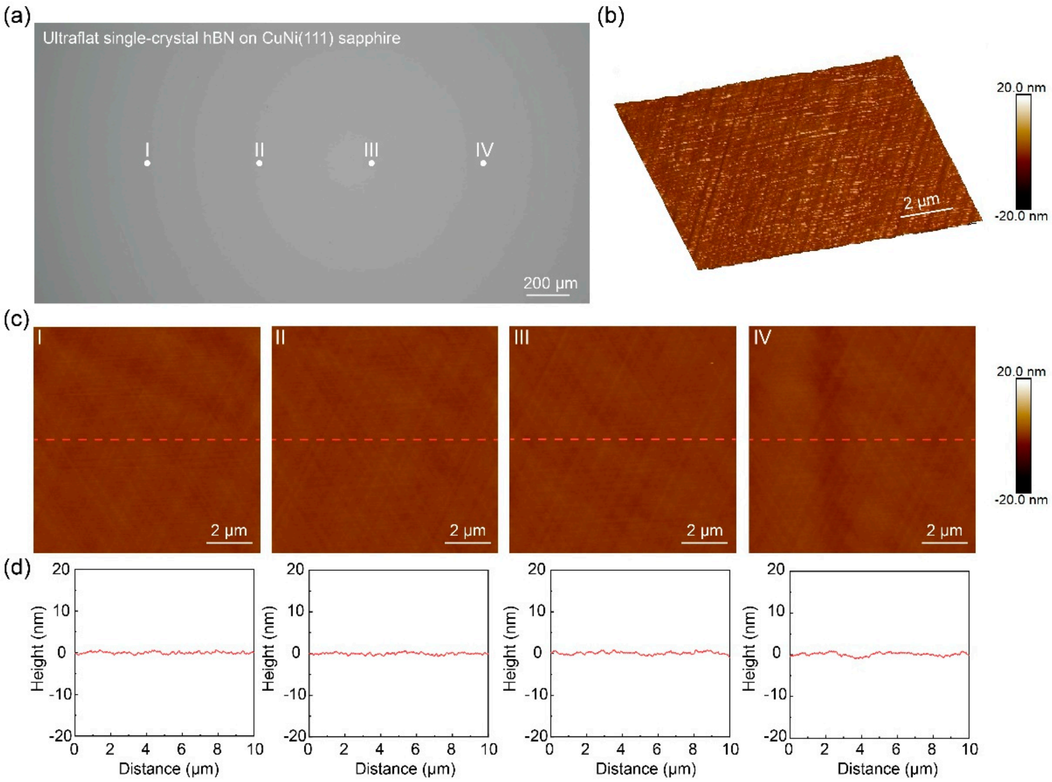

Figure. S2 Ultraflat single-crystal hBN on the CuNi(111)/sapphire wafer. (a) Optical microscopy image of USC-hBN on the CuNi(111)/sapphire. (b) 3D AFM image of USC-hBN on the CuNi(111)/sapphire. (c) Typical AFM images revealing the atomically flat hBN surface. (d) Height profiles along the red lines in (a), showing that the maximum height variations of hBN are ±1 nm at a lateral scale of 10 μm.

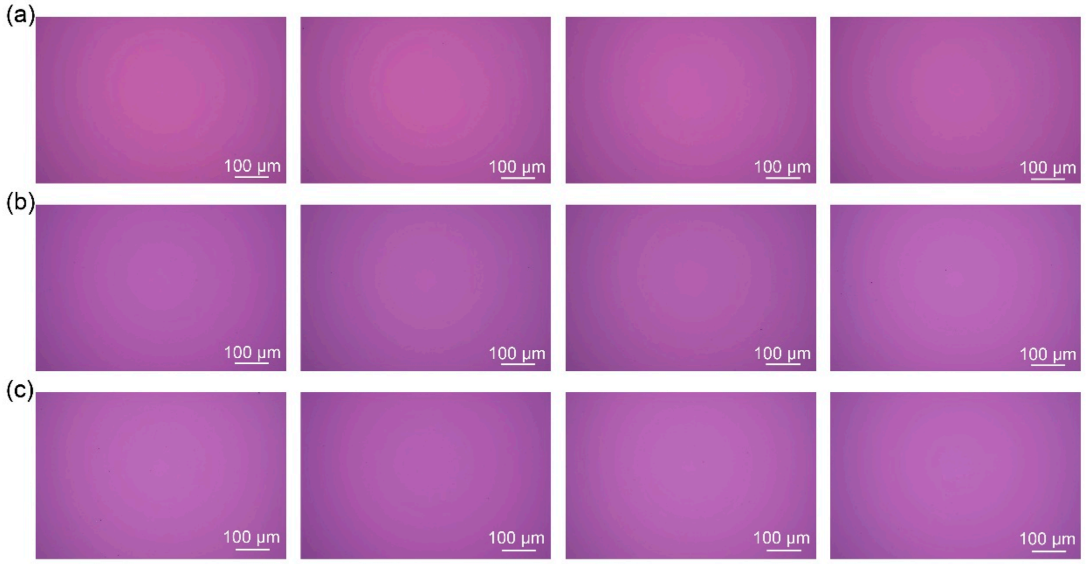

Figure. S3 Optical microscopy images of the stacked few-layer USC-hBN. (a) Optical microscopy images of monolayer USC-hBN. (b) Optical microscopy images of bilayer USC-hBN. (c) Optical microscopy images of trilayer USC-hBN. All samples exhibit clean and uniform surfaces, and no obvious wrinkles and cracks are observed.

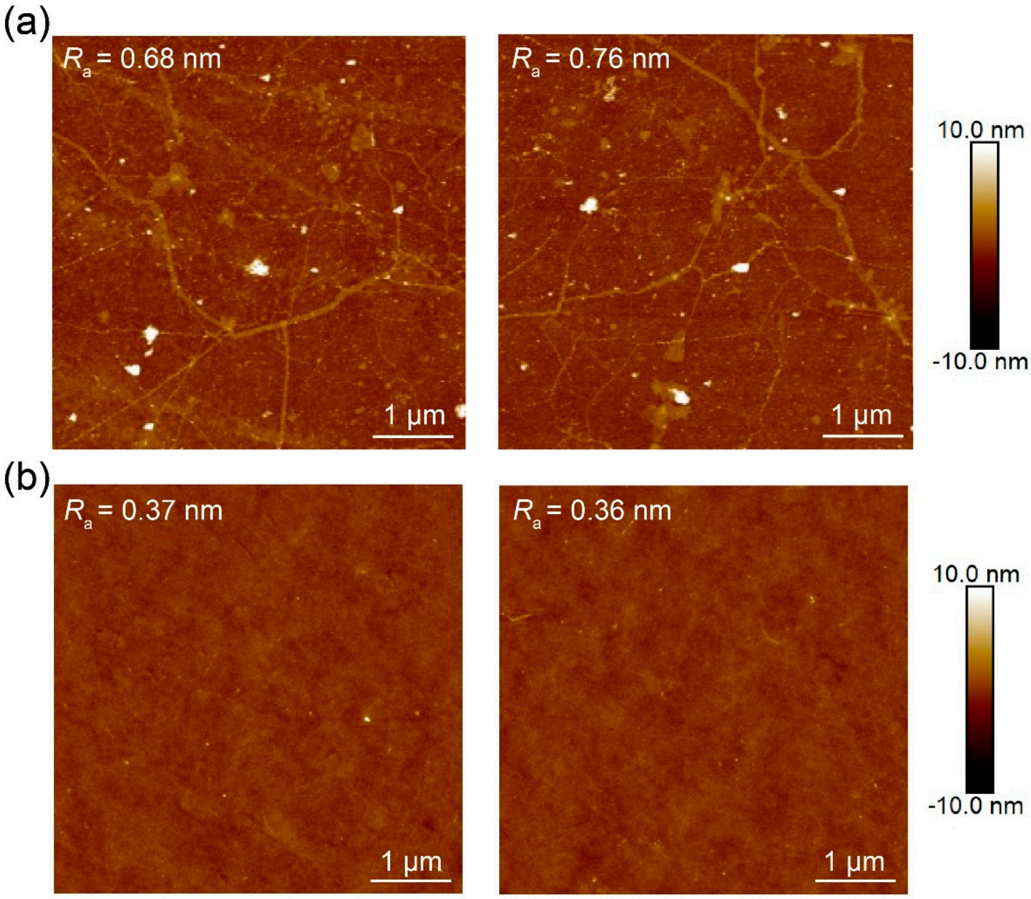

Figure. S4 AFM images of the stacked bilayer hBN. (a) AFM images of the stacked bilayer rough hBN (b) AFM images of the stacked bilayer USC-hBN.

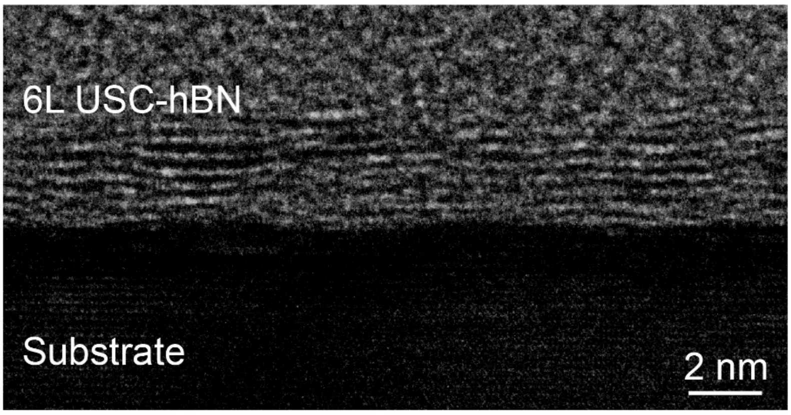

Figure. S5 Cross-sectional TEM image of stacked six-layer USC-hBN.

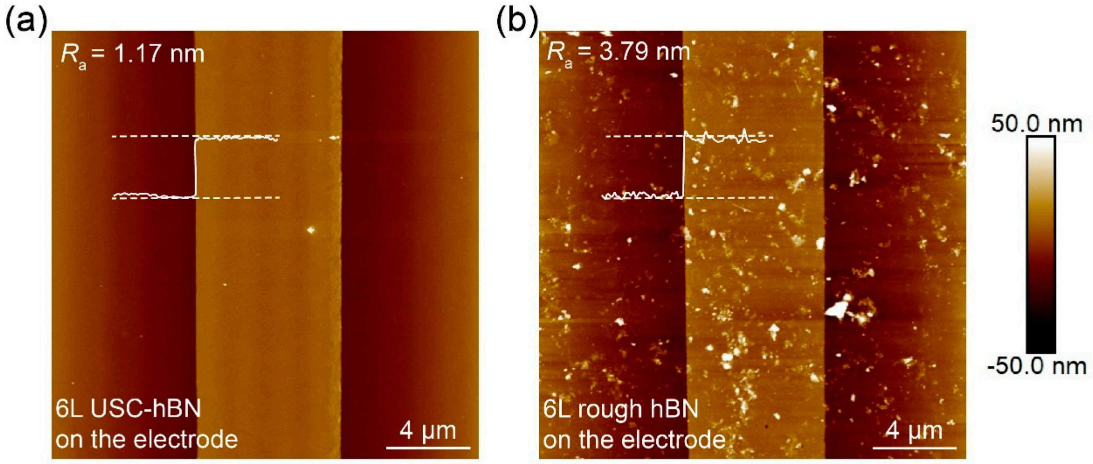

Figure. S6 AFM images of stacked six-layer hBN during memristor fabrication. (a) AFM image of six-layer USC-hBN on the electrode. (b) AFM image of six-layer rough hBN on the electrode. Compared with six-layer rough hBN, six-layer USC-hBN exhibits a cleaner and more uniform surface.

Table. S1 Benchmark comparison of representative hBN-based memristors.

No.	hBN type	hBN thickness	Wafer size	ON/OFF ratio	Reference
**1**	Exfoliated single-crystalline hBN	~4.5 nm, (~13–14 layers)	/	~10^3	*J. Semicond.***2022**, 43, 052003[26]
**2**	CVD-grown multilayer hBN	~8–10 nm, (~15–20 layers)	2*2cm^2	~10^2	*npj 2D Mater Appl* **2022**, 6, 50[4]
**3**	MOCVD-grown few-layer hBN	~2.08 nm, (~7–8 layers)	2 inch	~10	*ACS Appl. Mater. Interfaces* **2020**, 12, 46288–46295[24]
4	CVD-grown ultrathin hBN	~3 nm, (~9 layers)	/	~10^2	*Adv. Funct. Mater.* **2016**, 26, 2176–2184[7]
**5**	CVD-grown Ultraflat single-crystal hBN	Stacked 6 layers	4 inch	~10^3–10^4	This work

References

[26] Deng, Y.; Li, Y.; Wang, P.; Wang, S.; Pan, X.; Wang, D. Observation of resistive switching in a graphite/hexagonal boron nitride/graphite heterostructure memristor. *J. Semicond* 2022, 43(5), 052003. https://doi.org/10.1088/1674-4926/43/5/052003.

[4] Xie, J.; Afshari, S.; Sanchez Esqueda, I. Hexagonal boron nitride (h-BN) memristor arrays for analog-based machine learning hardware. *npj 2D Mater. Appl.* **2022**, *6*, 50. https://doi.org/10.1038/s41699-022-00328-2.

[24] Jeong, H.; Kim, J.; Kim, D.Y.; Kim, J.; Moon, S.; Ngome Okello, O.F.; Lee, S.; Hwang, H.; Choi, S.-Y.; Kim, J.K. Resistive switching in few-layer hexagonal boron nitride mediated by defects and interfacial charge transfer. *ACS Appl. Mater. Interfaces* **2020**, *12*, 46288–46295. https://doi.org/10.1021/acsami.0c12012.

[27] Qian, K.; Tay, R.Y.; Nguyen, V.C.; Wang, J.; Cai, G.; Chen, T.; Teo, E.H.T.; Lee, P.S. Hexagonal boron nitride thin film for flexible resistive memory applications. *Adv. Funct. Mater* **2016**, 26, 2176–2184. https://doi.org/10.1002/adfm.201504771.
